# Evaluation and Limitations of the Novel Chemiluminescent Enzyme Immunoassay Technique for Measuring Total Tau Protein in the Cerebrospinal Fluid of Patients with Human Prion Disease: A 10-Year Prospective Study (2011–2020)

**DOI:** 10.3390/diagnostics14141520

**Published:** 2024-07-15

**Authors:** Kong Weijie, Toshiaki Nonaka, Katsuya Satoh

**Affiliations:** 1 Division of Cellular and Molecular Biology, Nagasaki University Graduate School of Biomedical Sciences, Nagasaki City 852-8501, Japan; bb55324036@ms.nagasaki-u.ac.jp (K.W.); bb55417803@ms.nagasaki-u.ac.jp (T.N.); 2 Department of Health Sciences, Unit of Medical and Dental Sciences, Nagasaki University Graduate School of Biomedical Sciences, Nagasaki City 852-8523, Japan

**Keywords:** cerebrospinal fluid, Creutzfeldt–Jakob disease, diagnostic test, prion protein, chemiluminescent enzyme immunoassay, human prion disease

## Abstract

Background: Recently, the investigation of cerebrospinal fluid (CSF) biomarkers for diagnosing human prion diseases (HPD) has garnered significant attention. Reproducibility and accuracy are paramount in biomarker research, particularly in the measurement of total tau (T-tau) protein, which is a crucial diagnostic marker. Given the global impact of the coronavirus disease pandemic, the frequency of measuring this protein using one of the world’s fully automated assays, chemiluminescent enzyme immunoassay (CLEA), has increased. At present, the diagnosis and monitoring of neurological diseases mainly rely on traditional methods, but their accuracy and responsiveness are limited. There is limited knowledge of the accuracy of CLEA in tau measurements. We aimed to measure T-tau protein using CLEA and to elucidate its merits and limitations. Methods: We randomly selected 60 patients with rapidly progressive dementia, using ELISA and CLEA analysis of cerebrospinal fluid specimens. Additionally, we used Western blotting to detect the presence of 14-3-3 protein and employed real-time quaking-induced conversion (RT-QuIC) assays to analyze the same set of samples. Furthermore, we examined the correlation coefficient between ELISA and CLEA results in a subset of 60 samples. Moreover, using CLEA, we evaluated the diurnal reproducibility, storage stability, dilutability, and freeze–thaw effects in three selected samples. Results: In 172 patients, 172 samples were extracted, with each patient providing only one sample, and a total of 88 (35 men and 53 women) tested positive for HPD in the RT-QuIC assay. In contrast, all CSF samples from the remaining 84 patients without HPD (50 men and 34 women) tested negative in the RT-QuIC assay. Both ELISA and CLEA showed perfect sensitivity and specificity (100%) in measuring T-tau protein levels. In addition, ELISA and CLEA are similar in terms of measurement sensitivity and marginal effect of detection extrema. CLEA analysis exhibited instability for certain samples with T-tau protein levels exceeding 2000 pg/mL, leading to low reproducibility during dilution analysis. Conclusions: Our findings indicate that CLEA outperforms ELISA in terms of diurnal reproducibility, storage stability, and freeze–thaw effects. However, ELISA demonstrated superior performance in the dilution assay. Therefore, it is imperative to develop innovative approaches for the dilution of biomarker samples for CLEA measurements during clinical trials.

## 1. Background

Human prion disease (HPD), a fatal and incurable neurodegenerative disorder, is attributed to the misfolding of prion protein (PrP). Prion diseases encompass a phenotypically diverse spectrum of conditions characterized by aberrantly conformed protease-resistant proteins. These disorders can be classified into three types: sporadic forms (including Creutzfeldt–Jakob disease (CJD), sporadic fatal insomnia, and variably protease-sensitive prionopathy); genetic forms (including genetic CJD, fatal familial insomnia, and Gerstmann–Straussler–Scheinker syndrome); and acquired forms (including kuru, variant CJD, and iatrogenic CJD) [1].

In the past, the diagnosis of HPD relied on clinical manifestations and electroencephalogram (EEG) criteria [2] because of the inability to detect abnormal prion protein without a brain biopsy. Therefore, additional methods are required to diagnose HPDs. The clinical diagnosis is facilitated by the detection of biochemical markers in the patient’s cerebrospinal fluid (CSF). Western blotting is considered a reliable diagnostic tool for HPD because it enables the detection of total tau (T-tau) and 14-3-3 proteins in the CSF [3].

Enzyme-linked immunosorbent assay (ELISA) has been predominantly used to quantify Ab42, T-tau, and P-tau181 [4]. This technique involves manual or semi-automated addition and removal of reagents on a microplate using an ELISA processor. However, its widespread implementation in routine clinical laboratories has faced significant challenges [5]. It is important to highlight the lack of standardization and awareness regarding the pre-analytical importance and specimen management as well as analytical factors that may affect the final results. While this method has been used in non-neurological clinical settings with consistent detection measures in serum [6,7,8,9], there have been limited studies investigating its accuracy for biomarker detection in CSF and scarce research comparing methods and diagnostic performance on novel innovative platforms.

ELISA is a commonly used experimental technique, known as enzyme-linked immunosorbent assay. It detects and quantifies the presence or concentration of a target substance in a sample by exploiting the ability of antibodies to bind to specific molecules, such as proteins, cytokines, etc. This technology has a wide range of applications in medical diagnosis, biological research, and drug development. The CLEIA method is a chemiluminescence enzyme immunoassay. It is a highly sensitive experimental technique for the detection and quantitative analysis of specific substances in samples. This method uses enzyme-labeled antibodies to interact with the test substance and generates a fluorescence signal through a chemical reaction. The CLEIA method has a wide range of applications in medical diagnosis, environmental monitoring, food safety, and other fields.

Despite being the most commonly employed technique for detecting core biomarkers in the CSF of patients with HPD, the ELISA method often results in substantial interlaboratory and intralaboratory variability, which limits their widespread adoption in clinical practice. To address this issue, a novel diagnostic approach was integrated with the ELISA detection system to improve the early diagnosis of HPD, which is crucial for effective disease prevention [10,11].

Since RT-QuIC has been confirmed in terms of accuracy and practicability in a long-term clinical application [7,8], we used the RT-QuIC method as a control to verify the reliability and accuracy of CLEA in this study.

Chemiluminescent enzyme immunoassay holds immense potential to revolutionize clinical practice and research, serving as a valuable indicator of normal or pathological biological processes and dynamic responses to therapeutic interventions. In a clinical setting, it can aid in the diagnosis, disease activity monitoring, and evaluation of treatment impact on clinical response. At the scientific research level, it provides crucial selection information for observational and experimental studies, while also serving as an alternative, secondary, or even primary endpoint in trials. While neurospecific liquid chemiluminescent enzyme immunoassays are currently lacking, the diagnosis and monitoring of neurological disorders rely heavily on neurological examinations, clinical assessments, outcome scoring, and neurophysiology; furthermore, peripheral nerve imaging techniques are continuously improving despite their limited functionality. Traditional methods offer a high degree of semi-quantification but lack accuracy when identifying potential abnormalities; they fail to differentiate between residual damage and active disease while exhibiting poor responsiveness. Specific fluid-based chemiluminescent enzyme immunoassays can simplify tasks such as diagnosing, predicting, and monitoring under active states. Moreover, with an increasing number of reported HPD cases emerging rapidly, there is an urgent need for sensitive and reliable biomarkers that personalize treatment approaches while enhancing cost-effectiveness [12]. The immunochromatographic assays and chemiluminescent enzyme immunoassays (CLEAs), produce faster results than polymerase chain reaction (PCR) [13,14]. We frequently use CLEAs for antigen and antibody testing because of their demonstrated utility. CLEAs provide advantages, such as simultaneous reproducibility, intraday reproducibility, and excellent storage stability.

By contrast, the approval of drugs for Alzheimer’s disease in the U.S. and Japan has increased the significance of using spinal fluid biomarkers to diagnose this condition. CLEA, which is known for its superior reproducibility compared to that of ELISA, can be employed to quantify CSF biomarkers [15].

We aimed to use CLEA to measure T-tau protein levels in CSF samples and demonstrate its utility for assessing tau levels in patients with HPD. In addition, we aimed to elucidate the strengths and weaknesses of CLEA, and we tried to show the feasibility of using CLEA to detect T-tau protein in cerebrospinal fluid.

## 2. Methods

### 2.1. Aim, Design, and Setting of the Study

In this 10-year prospective study (2011–2020), we aimed to demonstrate the utility of CLEA for assessing T-tau levels in the CSF samples of patients with HPD and elucidate its strengths and weaknesses. CSF biomarker analysis was conducted on 3000 patients with rapidly progressive dementia at Nagasaki University; then 172 samples were randomly selected from the 3000 patient samples for a more nuanced analysis.

### 2.2. Ethical Statement

The Ethics Committee of the Nagasaki University Graduate School of Biomedical Sciences approved the study protocol (ID No. UMIN000038398, UMIN000016855, and UMIN000003301). All patients or family members of patients provided written informed consent.

### 2.3. Patients

A total of 172 patients were randomly selected for further investigation. The diagnosis and subsequent analysis in all patients were validated by the Japanese Surveillance Commission. PrP genotyping was performed using genomic deoxyribonucleic acid (DNA) extracted from peripheral blood leukocytes.

Biochemical analysis of the CSF samples was conducted (T-tau protein assay kit (CLEA) Lumipulse^®^).

Imprecision and linearity of CLEA were tested using Lumipulse^®^ (Fujirebio Inc., Tokyo, Japan).

Imprecision was assessed by analyzing low- and high-quality control samples 10 times within the same analytical run (repeatability) or 30 times over a period of 15 days (reproducibility). The mean, standard deviation (SD), and coefficient of variation (CV) were calculated. For linearity evaluation, a CSF sample with a high concentration of T-tau protein was serially diluted using the diluent provided by the manufacturer (the raw data can be found in Table 1).

### 2.4. CLEA Measurement of T-Tau Protein in the CSF of 60 Patients

CLEA (T-tau protein assay kit Lumipulse^®^) was employed to quantify T-tau protein in the CSF according to the manufacturer’s instructions. The procedural steps involved in this analysis were as follows. The total tau calibrator or specimen, along with a biotinylated antibody solution, was added to the particle solution. Subsequently, the T-tau present in the specimens or calibrators specifically bound to the anti-T-tau monoclonal antibody (mouse) on the particles as well as to the biotinylated antibody (mouse). This binding led to the formation of biotinylated antibody–antigen immunocomplexes. The particles were thoroughly washed and rinsed to eliminate any unbound material. Alkaline phosphatase (ALP)-labeled streptavidin was selectively attached to the biotinylated immunocomplexes on the particles. A final round of washing and rinsing was performed to remove any remaining unbound materials that were mixed with the substrate solution, wherein the ALP-conjugated particles catalyzed the dephosphorylation of the adamantyl-1,2-dioxetane phosphate (AMPPD) present in the solution. This enzymatic reaction leads to the generation of luminescence at a maximum wavelength of 477 nm upon the cleavage of dephosphorylated AMPPD. The luminescent signal serves as an indicator for quantifying the T-tau protein levels across all samples.

### 2.5. ELISA of T-Tau Protein in the CSF of 60 Patients

Cerebrospinal fluid samples from all 60 patients were subjected to T-tau detection following the manufacturer’s instructions. The 14-3-3 proteins are a class of highly conserved proteins with multiple roles in cell signaling, and these proteins are abundantly expressed in the brain and have been detected in the cerebrospinal fluid of patients with different neurological disorders. T-tau detection is carried out using the 14-3-3c antibody. The analysis was performed simultaneously on four different plates (all from the same batch, n, 23J12AA), with an equal distribution of CJD and non-CJD samples in each plate. Protein Western blot signals were used to determine sample dilution empirically. Positive samples were diluted at a ratio of 1:20 using the provided dilution buffer, weak positive samples at a ratio of 1:5, and negative samples also at a ratio of 1:5. The diluted samples were then added to wells coated with specific antibodies against 14-3-3c and incubated for one hour at room temperature. After four wash steps, they were further incubated with an anti-14-3-3c detection antibody (diluted at a ratio of 1:100) according to the manufacturer’s instructions for another hour at room temperature. Following additional washing steps, an HRP-conjugated anti-IgG secondary antibody (diluted at a ratio of 1:100) was added and the samples were incubated for another hour at room temperature. Unbound HRP conjugates were washed away before reacting with substrate hydrogen peroxide-tetramethylbenzidine solution. The reaction was stopped by adding an acidic solution, and the absorbance of the resulting yellow product was measured using a microplate reader (Multiskan Ascent V 1.24) set to read absorbance values at 450 nm wavelength. A standard curve was generated by plotting the absorbance against T-tau concentration units using calibrator values, enabling the determination of arbitrary concentration units (AU/mL) for unknown samples based on this standard curve range between 125 and 16,000 AU/mL in human cerebrospinal fluid.

### 2.6. Biochemical Analysis of CSF Samples (T-Tau Protein by ELISA and 14-3-3 Protein Using Western Blotting and RT-QuIC Assay)

T-tau protein in the CSF was measured using ELISA following the manufacturer’s instructions. An identical standard was used for all the experiments. Western blotting was performed to measure the 14-3-3 protein levels, as previously described. The RT-QuIC assay was performed using recombinant human PrP by following established protocols. All CSF samples collected from patients suspected of having HPD were stored as aliquots at −80 °C until use. Assays were performed once every 2 weeks to minimize repeated freeze–thaw cycles. Hemorrhagic CSF samples were excluded from the study.

Samples were simultaneously tested six times at three concentrations (low, medium, and high) using the Lumipulse G600II system and the experimental reagent 6BX2101 for detecting T-tau protein levels. The obtained values for each sample were recorded to assess reproducibility. The coefficient of variation (CV) for the control group ranged from 1.8 to 3.7%, while the CV for the experimental group ranged from 1.2 to 2.3%. To evaluate day-to-day reproducibility, measurements were taken twice daily over five days by testing both control and experimental samples ten times at three concentrations (low, medium, high). In the control group, CV ranged from 2.6 to 3.2%, whereas for the experimental group, it ranged from 1.9 to 4%.

Reproducibility Experiment: Experimental Setup: lumipulse G600II. Experimental Reagent: 6BX2101. Target Test: T-tau protein. Reference Value: <2000 pg/mL Measurement Range: 141–2000 pg/mL.

The study design was to compare the reproducibility, stability, and effectiveness of CLEA and RT-QuIC as diagnostic methods for human viral diseases.

To compare the specificity, sensitivity, and predictive values of CLEA and RT-QuIC methods, we analyzed cerebrospinal fluid samples from 30 patients with prion disease and 30 control patients.

To assess the repeatability of CLEA and RT-QuIC methods, we simultaneously tested samples from 30 sCJD patients in a subset of 60 samples to examine the correlation coefficient between ELISA and CLEA results.

To evaluate the stability of CLEA and RT-QuIC methods, sCJD cerebrospinal fluid samples (n = 9) were incubated under short-term (0–7 days), medium-term (0–14 days), and long-term conditions (0–29 days) at extremely low temperature (−80 °C), low temperature (−20 °C), and room temperature (4 °C). We studied the detection values of tau protein using both detection methods [15].

Whole Blood Influence: To assess the impact of blood contamination on comparing CLEA and RT-QuIC in CSF samples, to investigate the influence of whole blood on T-tau measurements, two random samples were divided into three portions, resulting in a total of six experimental samples with varying percentages of whole blood (0, 1, and 10%). After thorough shaking, T-tau values were measured using both CLEA and ELCIA methods, revealing an increase in T-tau values ranging from 100 to 107% as blood volume increased.

We added varying proportions (0, 1, 10%) of blood cells into sCJD (n = 3) and control (n = 3) CSF samples. We studied the detection values of tau protein using both detection methods.

To investigate the effect of transportation storage on sample testing accuracy, freeze–thaw experiments were conducted on cerebrospinal fluid samples (n = 3), with multiple cycles performed for dissolution.

### 2.7. Statistical Analysis

Using IBM SPSS Statistics 25, statistical analysis was conducted on the sample test data. Using the statistical software, we carried out the normal distribution verification, the paired comparison, the multivariate test, and other methods to calculate the test data. The results are represented by a regression line and equation, wherein the intercept quantifies the constant error while the slope measures the proportional error. The 95% confidence intervals (CIs) of both the intercept and slope in the regression equation are employed to evaluate potential systematic differences and proportional disparities between these two methods. Furthermore, the slope and intercept elucidate whether values exhibit solely random discrepancies, thereby enabling consistent conclusions to be drawn from both methodologies. The results were as follows:

- In terms of reproducibility, the standard deviation (SD) ranged from 10.7 to 26.4, with a coefficient of variation (CV) ranging from 1.2 to 3.7%, consistently remaining below the threshold of 4.2.
- For future reproducibility, the SD values ranged from 10.1 to 31.7, with a CV range between 1.9 and 4.2%, indicating consistent CV values below the threshold of 5.3.
- Regarding storage stability at different temperatures, T-tau protein measurements remained relatively stable for up to 4 days at −20 °C and −80 °C but decreased by approximately 5% after storing for 29 days compared to measurements taken at 14 °C.
- Dilution capability varied among different types; however, most dilution ratios fell within the range of 43 to 49%, with deionized water being closest to a 50% dilution ratio.
- Resistance against blood contamination: even in cases where there is up to 10% whole blood contamination present, CLEIA detection remains highly sensitive in identifying cerebrospinal fluid samples with variations lower than CV (3%).

## 3. Results

### 3.1. Patient Profiles

Among the 172 patients, 88 (35 men and 53 women) were identified as having HPD by positive reactions in the RT-QuIC assay (see Supplementary Table S1a,b, Raw data S2). The remaining 84 patients (50 men and 34 women) were classified as non-HPD patients because of negative reactions in the RT-QuIC assay (Supplementary Table S1a,b, Raw data S2). Furthermore, we conducted a second-generation QuIC assay on all samples following the Additional Methods (Raw data S1), which yielded results consistent with our RT-QuIC assay findings [16]. The patients were diagnosed and categorized according to the new diagnostic criteria for sporadic HPD [17,18]. In 172 patients with 172 samples, we randomly selected 60 samples for analysis. The classification of these 45 patients with HPD was based on both the WHO criteria and the aforementioned new criteria, and they were confirmed as having typical and classical sporadic CJD. All patient diagnoses and subsequent analyses were validated by the Japanese Primary Disease Surveillance Commission.

### 3.2. Simultaneous Reproducibility of T-Tau Protein Measurements with CLEA in the Same Aliquots of the Control and Test Samples

The initial sample was partitioned into six distinct aliquots for simultaneous measurements across three control and test samples. The SD ranged from 10.7 to 26.4, signifying that fluctuations exceeding a magnitude of 1000 pg/mL in T-tau protein measurements by CLEA within this range of SD values were observed among the samples. Furthermore, the CV ranged from 1.2 to 3.7%, consistently maintaining levels below the threshold of 4% (Table 1).

### 3.3. Stability of T-Tau Protein CLEA Measurements in the Control and Test Samples

One sample was injected in 10 replicates and measured twice daily at different time points, whereas the T-tau measurements were assessed over an additional 5-day period (Table 2). SD values ranged from 10.1 to 31.7, indicating some fluctuations of >1000 pg/mL in T-tau CLEA measurements. Furthermore, the CV ranged from 1.9 to 4.2%, indicating that the CV values were consistent and below the threshold of 5% [19].

### 3.4. Storage Stability of CSF Samples

**Figure 1:**
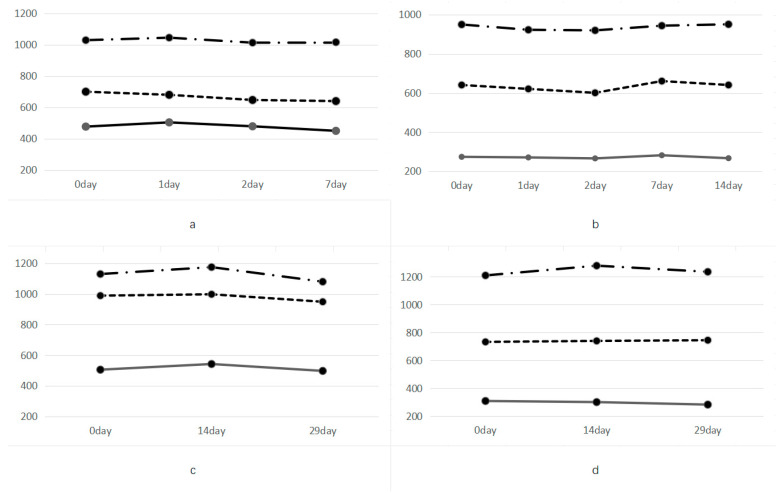
(**a**–**d**) Storage stability of CSF samples. The 500 uL CSF samples from all the patients are divided into five 100 uL aliquots. T-tau protein levels decline after 2 days of storage at room temperature, with an approximate deactivation rate of 6–9% within a week under the same conditions. T-tau protein concentrations remain relatively stable in samples stored at 4 °C, showing only around a 3% decrease compared to those stored at 14 °C. T-tau protein levels measured in samples stored at −20 °C and −80 °C remain nearly unchanged over a period of 14 days but demonstrate around a 5% decrease after storage for up to 29 days. Considering the results obtained from multiple samples with concentrations exceeding 1000 pg/mL, we infer that any variations in the T-tau protein measurements between samples stored at −20 °C and −80 °C fall within the margin of error. CSF, cerebrospinal fluid. Every line of the same shape is from the same random sample. A total of 3 samples were randomly selected, and the three random samples were divided into 4 equal parts, with each abcd having one part. The abcd diagram represents the experimental results at room temperature, the experimental results at 4 degrees, the experimental results at minus 20 degrees, and the experimental results at minus 80 degrees.

The 500 µL CSF sample from all patients was divided into five 100 µL samples. After 2 days of storage at room temperature, T-tau levels decreased, with an inactivation rate of about 6–9% within a week under the same conditions (Figure 1). Compared with the samples stored at 14 °C, in the samples stored below 4 °C, T-tau protein concentration fell by about 3%. The T-tau levels measured in samples stored at −20 °C and −80 °C remained almost constant for 14 days but showed a decrease of about 5% after up to 29 days (Figure 1).

### 3.5. Reproducibility of the Results with T-Tau Protein Dilution in CSF Samples

We assessed the reproducibility of the results by diluting the T-tau protein samples, as well as by diluting the T-tau protein in the CSF samples, using various solutions to achieve a 50% reduction in the T-tau protein concentration (Table 3). Most dilution ratios ranged from 43 to 49%, with the dilution ratio resulting from deionized water being the closest to 50%. Although deionized water is considered a better, yet not optimal, dilution solution, we serially diluted the CSF samples multiple times using deionized water (Table 3). These findings suggest that with each dilution, the data exhibit smoother and less pronounced patterns (Table 3). In conclusion, serial dilutions are unsuitable for determining T-tau protein levels using CLEA.

### 3.6. Similarities between ELISA and CLEA

**Figure 2:**
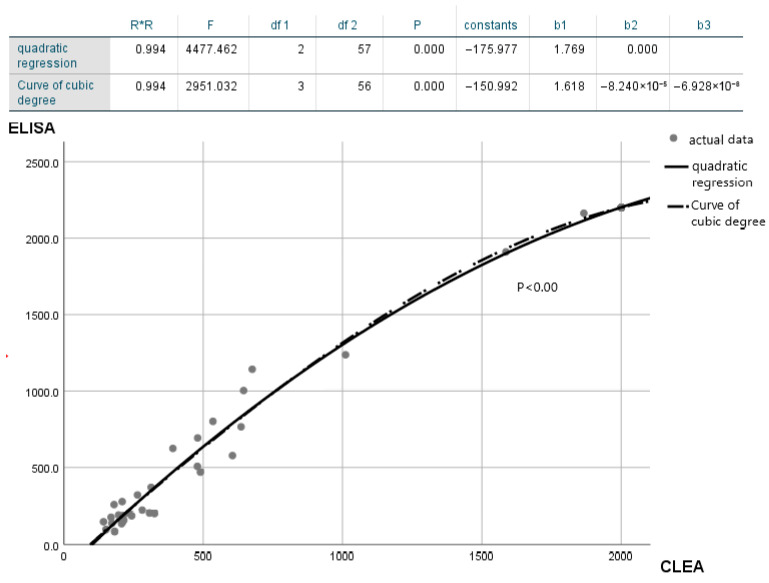
Relationship between T-tau protein levels measured using ELISA and CLEA. ROC analysis is performed using Lumipulse to investigate the diagnostic performance between CLEA and ELISA and evaluate the ROC analysis of the new automated method. According to the detection results, there is a strong correlation between CLEA and ELISA, indicating that both detection methods can effectively detect T-tau protein. CLEA, chemiluminescent enzyme immunoassay; ELISA, enzyme-linked immunosorbent assay; ROC, receiver operating characteristic.

The results of the study showed that, in all samples with T-tau levels exceeding 2000 pg/mL measured using ELISA, the CLEA also measured T-tau levels exceeding 2000 pg/mL. In patients with HPD, T-tau levels as measured by CLEA were consistently higher than 1300 pg per milliliter. However, in patients without HPD, this level was below 1300 pg/mL. By comparing the CLEAs measured with ELISA T-tau protein levels, we found that, in the same sample, the resulting value is higher. There was a strong correlation between ELISA and CLEA results (Figure 2) (*p* < 0.000), as detailed in Supplementary Table S1. Because many patients had T-tau levels above 2000 pg/mL at the time of the CLEA measurement and no HPD patient reached 1011 pg/mL, an ELISA threshold set at 1300 pg/mL could not be used to determine a specific cutoff point. Using the most sensitive CLEA value (1011 pg/mL) as the reference corresponding to the ELISA cutoff point (1300 pg/mL), the corresponding CLEA value was approximately 936 pg/mL. For further statistical verification of this correlation, an ANOVA was performed (Supplementary Table S2).

### 3.7. Results of Biomarker Measurements in the CSF of All Patients

The sensitivity and specificity results of the biomarker measurements in all patients are presented in Table 4, and the detailed biomarker data for all patients are shown in Supplementary Table S1. A positive reaction was seen in both the first- and second-generation RT-QuIC assays in all patients with sporadic HPD, whereas a negative reaction was seen in both assays in all patients without HPD. All patients with Huntington’s disease (HD) tested positive for the presence of 14-3-3 protein measured using Western blotting and for T-tau protein measured using ELISA (1300 pg/mL). Conversely, patients without sporadic HPD tested negative for 14-3-3 protein measured using Western blotting and T-tau protein measured using ELISA (<1300 pg/mL). Both the sensitivity and specificity of T-tau protein measurements with CLEA were 100%.

The original sample was divided into six aliquots that were assayed simultaneously. These samples were analyzed in triplicate in both the control and HPD cases. The SD values ranged from 10.7 to 26.4, indicating that there were fluctuations of >1000 pg/mL in the T-tau protein levels measured by CLEA. Conversely, the CV values ranged from 1.2 to 3.7%, suggesting that the CV remained constant at <4% (Table 1) (see Supplementary Figure S1).

Mochelay’s sphericity test is a method used in statistics to assess the sphericity of a data set or whether the data points are evenly distributed in multidimensional space. It can help us to determine whether the data set is clustering or moving toward a direction of distribution, and so on and so forth. By performing the Mochi sphericity test, we can better understand and describe the characteristics of the data and their distribution.

Concurrently, we conducted additional testing using Mauchly’s sphericity test and performed a conditional operation of Mauchly’s sphericity test using statistical methods. Upon verifying the normality (see Supplementary Table S3), the *p*-values of all data were >0.05. Subsequently, Mochelay’s sphericity test was performed (see Supplementary Table S3). Owing to the violation of the spherical hypothesis with a Greenhouse–Gessler value of 0.398, an epsilon (ε) correction was required. Since epsilon (ε) was <0.75, we employed the Greenhouse–Gessler method. In the time-period correlation comparison, the Greenhouse-Geissler *p*-value was calculated as 0.182, with an observed significance level of *p* > 0.05, indicating no difference in ELISA results between the experimental and control groups at different time points. In the time x group analysis, the calculated Greenhouse-Geissler *p*-value was 0.348, with an observed significance level of *p* > 0.05 (see Supplementary Table S4), suggesting no difference in ELISA detection results between the experimental and control groups at different time points. Furthermore, by examining the contour line (see Supplementary Figure S2), it was possible to determine whether there was a noticeable interaction. Both the control and experimental groups exhibited significant changes over time with consistent trends, as indicated by the parallel line segments. This outcome aligns well with previously computed results.

Therefore, we can conclude that the CLEA detection method demonstrated high accuracy in simultaneously measuring T-tau levels in the test and control samples during reproducibility experiments. T-tau protein measurements were performed twice daily in 10 consecutive samples, with each sample injected once. Additionally, T-tau protein levels were measured using the same method for an additional 5 days (Table 2). The SD values ranged from 10.1 to 31.7, indicating a fluctuation of >1000 pg/mL in T-tau protein levels measured by CLEA. Moreover, the CV ranged from 1.9 to 4.2%, suggesting consistent CV values of <5 (see Supplementary Figure S3).

Concurrently, we employed Mauchly’s sphericity test to perform time-period comparisons, intergroup comparisons, and an analysis of interactions between time periods and groups. First, a map of outliers visually displayed the degree of offset (CV%). This illustration demonstrates the reproducibility of diurnal differences in CLEA detection accuracy between the experimental and control groups. We aimed to determine whether the accuracy of CLEA was affected by the interactions between time and group using Mauchly’s sphericity test. Initially, data normality was verified using calculations (see Supplementary Table S5), yielding a result of *p* > 0.1, which aligned with the normal distribution assumption. Subsequently, variance homogeneity was assessed, and the results indicated that the fluctuation of F values at the 10 time points ranged from 0.457 to 1.103 (*p* ranging from 0.353 to 0.536). The quality of variance was satisfied at each time point.

The assumptions for employing Mauchly’s sphericity test were satisfied, enabling us to use this test to assess the impact of the interaction between groups and time periods on the accuracy of CLEA. Consequently, Mauchly’s sphericity test was conducted, and the results are presented in Supplementary Table S6. It is worth noting that Wend0.000 indicates an inability to determine the *p*-value as well as noncompliance with sphericity assumptions. Therefore, a direct analysis using the uncorrected method was not applicable; instead, an epsilon (ε) correction was necessary. Since our calculated epsilon (ε) value of 0.294 fell below 0.75, we employed a Greenhouse–Geisser correction for testing purposes (see Supplementary Table S6).

The resulting *p*-value from the Greenhouse–Geisser correction was 0.017, which is <0.05, signifying significant differences in reproducibility outcomes across different time points. On another note, it should be mentioned that the *p*-value for the time × group interaction was >0.05 (see Supplementary Table S7), indicating no substantial association between time points and interactive factors among groups; hence, there was no significant relationship between reproducibility outcomes at different time points.

The pairwise comparison results also indicated no significant correlation between the two groups at any time point. With a *p*-value > 0.05 at all time periods, there was no significant difference between groups 1 and 2 at any given time point (see Supplementary Table S8). Based on these findings, we can confidently conclude that CLEA detection accurately replicates diurnal variations in the experimental group compared with the control group.

As previously described, 500 µL cerebrospinal fluid samples from patients were tested in five 100 µL aliquots. After 2 days of storage at room temperature, T-tau levels decreased, and the inactivation rate was approximately 6–9% within 1 week under the same conditions (Figure 1). At 4 °C, T-tau levels remained relatively stable, showing only a decrease of approximately 3% compared to T-tau levels stored at 14 °C. T-tau levels in samples stored at −20 °C and −80 °C remained almost constant for 14 days but showed a decrease of approximately 5% after storage up to 29 days. It can be assumed that the changes in T-tau levels in samples stored at −20 °C and −80 °C are within the margin of error (Figure 1). SPSS Statistics 25 software (version 25) was used for statistical analysis of the experimental data. The F value fluctuated between 0.085 and 0.344, and the *p*-value fluctuated between 0.785 and 0.859 (Supplementary Table S2). It has also been demonstrated that the CLEA method has certain advantages in terms of the storage stability of CSF samples.

We assessed the reproducibility of measurements in the samples by diluting the T-tau protein samples, as well as by diluting the T-tau protein in the CSF samples, using various solutions to achieve a 50% reduction in the T-tau protein concentration (Table 3). Although the ratio of the dilution solution was close to 0.5 (50%), the actual dilution ratio ranged from 43 to 49%, with the ratio obtained through deionized water being the closest to 50%. Although deionized water is considered a better option, it is not an optimal dilution solution. Consequently, serial dilutions of the CSF samples were performed multiple times using deionized water (Table 3) (Supplementary Figure S4a,b). These findings suggest that increasing the data dilution leads to increased smoothing effects. In summary, serial dilutions are unsuitable for determining T-tau protein levels using CLEA.

The effect of blood contamination in the CSF samples of patients with sporadic HPD on the CLEA response was studied. Contamination with red blood cells can potentially affect the CLEA response, particularly during routine clinical examinations, where the inadvertent introduction of approximately 1 to 10% of blood into the sampler during lumbar puncture may lead to contamination of CSF samples. Therefore, it is imperative to establish well-defined sample processing guidelines prior to analysis. To assess this, we generated mixed samples by adding 1% and 10% of the whole blood to the CSF samples and evaluated their protein levels. Overall, our experimental findings demonstrate that even in the presence of up to 10% of whole blood contamination, CLEA detection remains highly sensitive in testing CSF samples with a CV of <3%. Conversely, as the quantity of blood in CSF samples increases, the accuracy of the conventional RT-QuIC method diminishes. When the contamination level exceeds 1250 blood cells/μL, the effectiveness of the RT-QuIC method declines [20] (Table 5).

The findings of this study support the feasibility of using the new CLEA technique to detect total tau protein in the CSF of patients with HPD. On comparing ELISA and CLEA, CLEA resulted in superior daily reproducibility, storage stability, and freeze–thaw performance, whereas ELISA performed better in dilution tests. Therefore, the development of novel CLEA dilution solutions for future clinical trials is imperative. The experimental evidence supporting these findings was obtained through the analysis of CSF samples from 60 randomly selected patients. In addition, ELISA and CLEA are similar in terms of measurement sensitivity and the marginal effect of detection extrema (Figure 2).

We must acknowledge the limitations of our study. First, the relatively small sample size may restrict the generalizability of these findings. Second, the homogeneity within our sample, consisting solely of individuals from a single ethnic subgroup, could introduce demographic variability; however, we made efforts to mitigate this influence through mixed modeling. The determination of biomarkers in CSF has demonstrated significant practicality in predicting pathology. Rapid, appropriate, and sensitive detection could benefit serological research and decision-making. However, the standardization and simplification of biomarker measurement methods remain fundamental challenges in clinical practice. The pre-analytical and analytical variability of biomarkers for diagnosing human prion disease and related dementias impede their widespread use in routine and clinical settings as well as the establishment of universally recognized thresholds. Nevertheless, a simplified method that does not require specialized equipment such as ELISA would facilitate its extensive utilization at any location.

## 4. Conclusions

Here we demonstrate the superiority of the new CLEA method in terms of automation, surpassing even our standard ELISA for T-tau and 14-3-3 protein detection within our study cohort. In conclusion, our findings underscore the robustness and clinical utility of CLEA for analyzing CSF samples as a valuable diagnostic tool for HPDs, warranting its inclusion in the comprehensive evaluation of patients presenting with human prion disease.

## Figures and Tables

**Table 1:** Simultaneous reproducibility in the same aliquots of the control and test samples.

	Control Samples			Test Samples		
	Sample 1	Sample 2	Sample 3	Sample 4	Sample 5	Sample 6
1	311	511	822	452	660	1246
2	320	535	855	440	693	1249
3	330	533	854	444	685	1230
4	342	546	829	450	707	1299
5	325	539	861	457	687	1242
6	346	532	857	446	708	1213
Number	6	6	6	6	6	6
Min	311	511	822	440	660	1213
Max	346	546	861	457	708	1299
Mean	329.0	532.7	846.3	448.2	690.0	1246.5
SD	12.1	10.7	15.0	5.6	16.1	26.4
CV (%)	3.7	2.0	1.8	1.2	2.3	2.1

Control samples: the content of T-tau protein in all samples was detected by ELISA. Test samples: the content of T-tau protein in all samples was detected by CLEA. Abbreviations: SD, standard deviation; CLEA, chemiluminescent enzyme immunoassay; CV, coefficient of variation; ELISA, enzyme-linked immunosorbent assay.

**Table 2:** Stability of the T-tau protein CLEA measurements.

	Control Samples			Test Samples		
	Aliquot 1	Aliquot 2	Aliquot 3	Aliquot 4	Aliquot 5	Aliquot 6
Day 1-1	302	515	817	347	877	1066
Day 1-2	307	512	843	390	916	1157
Day 2-1	315	520	818	346	884	1114
Day 2-2	320	515	793	347	897	1092
Day 3-1	312	488	805	348	855	1067
Day 3-2	321	496	824	374	892	1107
Day 4-1	333	536	833	372	876	1119
Day 4-2	333	543	876	380	897	1103
Day 5-1	308	535	829	353	913	1152
Day 5-2	327	531	836	361	889	1154
n	10	10	10	10	10	10
Min	302	488	793	346	855	1066
Max	333	543	876	390	916	1157
Mean	317.8	519.1	827.4	361.8	889.6	1113.1
SD	10.3	16.8	21.5	15.3	17.1	31.7
CV (%)	3.2	3.2	2.6	4.2	1.9	2.9

Control samples: the content of T-tau protein in all samples was detected by ELISA. Test samples: the content of T-tau protein in all samples was detected by CLEA. Abbreviations: SD, standard deviation; CLEA, chemiluminescent enzyme immunoassay; CV, coefficient of variation; ELISA, enzyme-linked immunosorbent assay.

**Table 3:** (**a**) Reproducibility of T-tau protein measurements on diluting the CSF samples. Results on diluting the tau protein sample by 1/2 dilution using three solutions (PBS, pure water, and the dissolving solution contained in the kit). (**b**) Results of stage dilution of T-tau protein in CSF samples using pure water.

(**a**)				
	Total tau protein measured using CLEA without dilution	1/2 dilution with PBS	1/2 dilution with pure water	1/2 dilution with dissolving solution contained in the kit
#1	533	240 (0.450)	250 (0.469)	243 (0.456)
#2	628	295 (0.470)	301 (0.479)	308 (0.490)
#3	629	273 (0.434)	289 (0.459)	287 (0.456)
(**b**)				
	Total tau protein measured using CLEA without dilution	1/2 dilution with pure water	1/4 dilution with pure water	1/8 dilution with pure water
#4	1806	875 (0.484)	416 (0.230)	177 (0.098)
#5	1826	892 (0.488)	419 (0.229	187 (0.102)
#6	1876	915 (0.484)	435 (0.232)	187 (0.100)

The ratio of tau protein measured at different dilutions with the undiluted solution set to 1 is included within parenthesis. The ratio of tau protein measured at 1/2 dilution when the undiluted solution was set to 1. Abbreviations: PBS, phosphate-buffered saline; T-tau, total tau; CLEA, chemiluminescent enzyme immunoassay; CSF, cerebrospinal fluid. The ratio of tau protein measured at different dilutions with the undiluted solution set to 1 is included within parenthesis. The ratio of tau protein measured at different dilutions with the undiluted solution set to 1. Abbreviations: T-tau, total tau; CLEA, chemiluminescent enzyme immunoassay; CSF, cerebrospinal fluid. #, group.

**Table 4:** Comparison of biomarkers from various detection methods, including their sensitivity and specificity.

	RT-QuIC Assay		14-3-3 Protein by Western Blotting	Total Tau Protein by ELISA	Total Tau Protein by CLEA
	First Generation	Second Generation		(Cutoff: 1300 pg/mL)	(Cutoff: 1000 pg/mL)
Sensitivity	100%	100%	100%	100%	100%
Specificity	100%	100%	100%	100%	97.8%

Abbreviations: RT-QuIC, real-time quaking-induced conversion; ELISA, enzyme-linked immunosorbent assay; CLEA, chemiluminescent enzyme immunoassay. CLEA cutoff: 1000; the detection limit value of the instrument, Lumipulse G600II, for T-tau protein is 1000. This study involved about 172 rapidly progressing neurodegenerative disease patients with cerebrospinal fluid samples, including a random sampling of 60 biomarkers.

**Table 5:** Effect of blood contamination on CLEA responses in CSF samples.

Group		t0	t1	t2
1		694	682	739
2		678	705	738
		t0	t1	t2
N	valid	2	2	2
	invalid	0	0	0
Mean		686	693.5	738.5
SEM		8	11.5	0.5
Median		686	693.5	738.5
SD		11.314	16.263	0.707
CV (%)		1.649	2.345	0.0001

Group: group 1 and group 2 were randomly extracted cerebrospinal samples, and the amount of T-tau protein in the sample was added to the sample using the CLEA detection technique. Abbreviations: CLEA, chemiluminescent enzyme immunoassay; CSF, cerebrospinal fluid; SEM, standard error of the mean; SD, standard deviation; CV, coefficient of variation.

## Data Availability

The data were obtained from the patient’s attending physician and the Japan Prion Disease Surveillance Committee at the time of the investigation and pathological autopsy. All raw data generated in this study were submitted for review as Raw data S1–S3.

## Supplementary Materials

The following supporting information can be downloaded at: https://www.mdpi.com/article/10.3390/diagnostics14141520/s1, Supplementary Table S1. Summary of the CSF biomarkers in SST person. (a). Summary of CSF biomarkers in person with sporadic human prion disease. (b) Summary of CSF biomarkers in individuals without sporadic human prion disease. Supplementary Table S2. Relationship between T-tau protein levels measured using ELISA and CLEIA. Supplementary Table S3 (a). Mauchly’s sphericity test conditional validation. (b). Levin equivalence test for error variance. Supplementary Table S4. Within-subject effect test. Supplementary Table S5. Verification of the normal distribution of diurnal reproducibility (normality test). Supplementary Table S6. Mauchly’s sphericity test. Supplementary Table S7. Within-subject effect test. Supplementary Table S8. Pair comparison. Supplementary Figure S1. Simultaneous reproducibility of T-tau protein CLEIA measurements in the same aliquots of control and CSF samples (% CV). Supplementary Figure S2. Estimation of the marginal mean. Supplementary Figure S3. Diurnal reproducibility of T-tau protein CLEIA measurements in the same aliquots of the control and test samples. Supplementary Figure S4. (a). Reproducibility of results with T-tau protein dilution in CSF samples. (b). Reproducibility of results with T-tau protein dilution in CSF samples.

## Abbreviations

ALP	alkaline phosphatase
AUC	relative area under the curve
CI	confidence interval
CJD	Creutzfeldt–Jakob disease
CLEA	chemiluminescent enzyme immunoassay
CNS	central nervous system
CSF	cerebrospinal fluid
CV	coefficient of variation
Diff	difference
DNA	deoxyribonucleic acid
EEG	electroencephalogram
ELISA	enzyme-linked immunosorbent assay
FFI	fatal familial insomnia
gCJD	genetic CJD
HPD	human prion disease
NPV	negative predictive value
PBS	phosphate-buffered saline
PCR	polymerase chain reaction
PPV	positive predictive value
prion PrP	protein
PrPC	cellular prion protein
PrPSc	scrapie prion protein
Rcf	relative centrifugal force rel.
Ref	reference
Rfu	relative fluorescence units
ROC	receiver operating characteristic
Rpm	revolutions per minute
RT-QuIC	real-time quaking-induced conversion
sCJD	sporadic CJD
SD	standard deviation
SEM	Standard error of the mean
