# Supplementary material for: Evaluation and Limitations of the Novel Chemiluminescent Enzyme Immunoassay Technique for Measuring Total Tau Protein in the Cerebrospinal Fluid of Patients with Human Prion Disease: A 10-Year Prospective Study (2011–2020)
Source: Diagnostics (Basel). 2024 Jul 15;14(14):1520. doi: 10.3390/diagnostics14141520 (PMC11275853; doi:10.3390/diagnostics14141520)
Supplement: Supplementary file 1 [file diagnostics-14-01520-s001.zip › Supplementary Tables S1-S8.pdf]

Supplementary Table S1ab. Cerebrospinal fluid biomarkers in patients with brain diseases

S1a. Summary of CSF biomarkers in person with sporadic human prion disease

Sex	14-3-3 protein by western blotting	generation QuIC assay	generation QuIC assay	T-tau protein by ELISA (pg/)	T-tau protein by CLEIA (pg/)
male	positive	positive	positive	>2,200	>2,000
male	positive	positive	positive	1,910	1586
	positive	positive	positive	>2,200	>2,000
male	positive	positive	positive	>2,200	>2,000
male	positive	positive	positive	>2,200	>2,000
male	positive	positive	positive	>2,200	>2,000
male	positive	positive	positive	>2,200	>2,000
male	positive	positive	positive	>2,200	>2,000
male	positive	positive	positive	>2,200	>2,000
male	positive	positive	positive	>2,200	>2,000
male	positive	positive	positive	>2,200	>2,000
male	positive	positive	positive	1,967	1,727
male	positive	positive	positive	>2,200	>2,000
male	positive	positive	positive	2180	>2,000
male	positive	positive	positive	>2,200	>2,000
male	negative	positive	positive	1,870	1585
male	positive	positive	positive	>2,200	>2,000
male	positive	positive	positive	>2,200	>2,000
male	positive	positive	positive	>2,200	>2,000
male	positive	positive	positive	2,163	1,866
male	positive	positive	positive	>2,200	>2,000
male	positive	positive	positive	>2,200	>2,000
female	positive	positive	positive	>2,200	>2,000
female	positive	positive	positive	>2,200	>2,000
female	positive	positive	positive	>2,200	>2,000
female	positive	positive	positive	>2,200	>2,000
female	positive	positive	positive	>2,200	>2,000

female	positive	positive	positive	>2,200	>2,000
female	positive	positive	positive	>2,200	>2,000
female	positive	positive	positive	>2,200	>2,000
female	positive	positive	positive	>2,200	>2,000
female	positive	positive	positive	1,966	1,307
female	positive	positive	positive	>2,200	>2,000
female	positive	positive	positive	>2,200	>2,000
female	positive	positive	positive	2,101	>2,000
female	positive	positive	positive	>2,200	>2,000
female	positive	positive	positive	>2,200	>2,000
female	positive	positive	positive	>2,200	>2,000
female	positive	positive	positive	>2,200	>2,000
female	positive	positive	positive	>2,200	>2,000
female	positive	positive	positive	2,117	>2,000
female	positive	positive	positive	>2,200	>2,000
female	positive	positive	positive	>2,200	>2,000
female	positive	positive	positive	>2,200	>2,000
female	positive	positive	positive	>2,200	>2,000

S1b. Summary of CSF biomarkers in without sporadic human prion disease

Sex	14-3-3 protein by western blotting	generation QuIC assay	generation QuIC assay	T-tau protein by ELISA (pg/ml)	T-tau protein by CLEIA (pg/ml)	Disease
male	negative	negative	negative	1142.5	676	primary malignant lymphoma
male	negative	negative	negative	82.5	182	drug toxic encephalopathy
male	negative	negative	negative	174	169	autoimmune encephalopathy
male	negative	negative	negative	201.7	233	alcohol-dependent LCCA
male	negative	negative	negative	145	142	hypoxic encephalopathy
male	negative	negative	negative	693	480	hypoxic encephalopathy
male	negative	negative	negative	342	370	epilepsy
male	negative	negative	negative	157	208	DLB
male	negative	negative	negative	169	173	epilepsy
male	negative	negative	negative	356	450	epilepsy
male	negative	negative	negative	155	127	hypoxic encephalopathy
male	negative	negative	negative	155	215	MSA-C
male	negative	negative	negative	276.5	209	alcohol-dependent LCCA
male	negative	negative	negative	257	180	metabolic encephalopathy
male	negative	negative	negative	184	243	DLB
male	negative	negative	negative	187	228	epilepsy
male	negative	negative	negative	95	151	autoimmune encephalopathy
male	negative	negative	negative	578	605	alcohol-dependent LCCA
male	negative	negative	negative	394	602	Alzheimer's disease
male	negative	negative	negative	221	281	COVID-19 syndrome
male	negative	negative	negative	141	210	SCA
male	negative	negative	negative	423	381	steroid-responsive encephalopathy
female	negative	negative	negative	370	313	epilepsy
female	negative	negative	negative	402	430	epilepsy
female	negative	negative	negative	852	737	epilepsy
female	negative	negative	negative	186	213	epilepsy
female	negative	negative	negative	181	194	epilepsy
female	negative	negative	negative	109	104	epilepsy

female	negative	negative	negative	189	196	epilepsy
female	negative	negative	negative	820	585	epilepsy
female	negative	negative	negative	624.5	391	epilepsy
female	negative	negative	negative	132.1	207	epilepsy
female	negative	negative	negative	320.2	264	epilepsy
female	negative	negative	negative	1003.1	645	epilepsy
female	negative	negative	negative	801.4	535	epilepsy
female	negative	negative	negative	283	319	epilepsy
female	negative	negative	negative	202	307	steroid-responsive encephalopathy
female	negative	negative	negative	766	636	epilepsy
female	negative	negative	negative	1205	907	steroid-responsive encephalopathy
female	negative	negative	negative	198	325	epilepsy
female	negative	negative	negative	132	181	epilepsy
female	negative	negative	negative	469	490	steroid-responsive encephalopathy
female	negative	negative	negative	1237	1011	steroid-responsive encephalopathy
female	negative	negative	negative	507	479	steroid-responsive encephalopathy
female	negative	negative	negative	172	135	steroid-responsive encephalopathy

Abbreviations: RT-QuIC, real-time quaking-induced conversion; ELISA, enzyme-linked immunosorbent assay; CLEIA, chemiluminescent enzyme immunoassays; LCCA left common carotid artery; DLB Lewy Body Dementia; MSA-C multiple system atrophy c type; SCA Spinocerebellar ataxia; COVID-19 Coronavirus disease 2019；

Supplementary Table S2. Relationship between t-tau protein levels measured ELISA and CLEIA

ANOVA						
		Sum of squares	DF	Meansquare	F	Sig
t0	MSB	96644.917	3	32214.972	0.257	0.855
	MSE	1003824.000	8	125478.000		
	SST	1100468.917	11			
t1	MSB	136660.667	3	45553.556	0.344	0.794
	MSE	1058743.333	8	132342.917		
	SST	1195404.000	11			
t2	MSB	94452.667	3	31484.222	0.251	0.859
	MSE	1003686.000	8	125460.750		
	SST	1098138.667	11			
t3	MSB	8214.000	1	8214.000	0.085	0.785
	MSE	386602.000	4	96650.500		
	SST	394816.000	5			

Abbreviations: ELISA, enzyme-linked immunosorbent assay; CLEIA, chemiluminescent enzyme immunoassays; t-tau, total tau protein; ANOVA Analysis of Variance; MSD Mean Square Displacement; MSEmean-square error; SST Sum of squares for total

Supplementary Table S3a. Mauchly's sphericity test conditional validation

Intrinsic effect	DF	Approximate square	DF	P	Epsilon[b]		
					Greenhouse-Geissler	Xin-ferdt	Lower-bound
time	0.000		14		0.399	0.992	0.200

S3b. Levin equivalence test for error variance

		F	DF 1	DF 2	sig P
t0	mean value	0.676	1	4	0.457
	median	0.144	1	4	0.723
	median and adjustment for degrees of freedom	0.144	1	3.201	0.728
	mean after shear	0.622	1	4	0.474
t1	mean value	0.566	1	4	0.494
	median	0.183	1	4	0.691
	median and adjustment for degrees of freedom	0.183	1	3.386	0.695
	mean after shear	0.532	1	4	0.506
t2	mean value	0.831	1	4	0.414
	median	0.244	1	4	0.648
	median and adjustment for degrees of freedom	0.244	1	3.158	0.654
	mean after shear	0.777	1	4	0.428
t3	mean value	1.103	1	4	0.353
	median	0.251	1	4	0.642
	median and adjustment for degrees of freedom	0.251	1	2.980	0.651
	mean after shear	1.016	1	4	0.371
t4	mean value	0.658	1	4	0.463
	median	0.189	1	4	0.686
	median and adjustment for degrees of freedom	0.189	1	3.352	0.690
	mean after shear	0.613	1	4	0.477
t5	mean value	0.652	1	4	0.465
	median	0.186	1	4	0.688
	median and adjustment for degrees of freedom	0.186	1	3.374	0.692
	mean after shear	0.607	1	4	0.480
t6	mean value	0.672	1	4	0.458
	median	0.235	1	4	0.653
	median and adjustment for degrees of freedom	0.235	1	3.282	0.658

	mean after shear	0.635	1	4	0.470
t7	mean value	0.457	1	4	0.536
	median	0.112	1	4	0.754
	median and adjustment for degrees of freedom	0.112	1	3.425	0.757
	mean after shear	0.423	1	4	0.551
t8	mean value	0.870	1	4	0.404
	median	0.251	1	4	0.643
	median and adjustment for degrees of freedom	0.251	1	3.103	0.650
	mean after shear	0.814	1	4	0.418
t9	mean value	0.758	1	4	0.433
	median	0.287	1	4	0.621
	median and adjustment for degrees of freedom	0.287	1	3.237	0.627
	mean after shear	0.719	1	4	0.444

We tested the null hypothesis that the error covariance matrix of the dependent variable after an orthogonal transformation would be proportional to the identity matrix.

[a] Design: [: cutoff:

Abbreviations: DF, degree of freedom

Supplementary Table S4. Within-subject effect test

		Sum of the squares III	DF	Mean square	F	P	Eta^2
Time	spherical degree	2562.556	5	512.511	2.123	0.105	0.347
	Greenhouse	2562.556	1.992	1286.191	2.123	0.182	0.347
	Xin-ferdt	2562.556	4.958	516.838	2.123	0.105	0.347
	lower-bound	2562.556	1	2562.556	2.123	0.219	0.347
Time * group	spherical degree	1457.222	5	291.444	1.208	0.342	0.232
	Greenhouse	1457.222	1.992	731.405	1.208	0.348	0.232
	Xin-ferdt	1457.222	4.958	293.905	1.208	0.342	0.232
	lower-bound	1457.222	1	1457.222	1.208	0.334	0.232
Deviation (time)	spherical degree	4827.222	20	241.361			
	Greenhouse	4827.222	7.969	605.717			
	Xin-ferdt	4827.222	19.833	243.399			
	lower-bound	4827.222	4	1206.806			

Abbreviations: DF, degree of freedom

Supplementary Table S5. Verification of the normal distribution of diurnal reproducibility (normality test)

0		Kolmogorov–Smirnov[a]			Shapiro–Wilk		
		Value	DF	P	Value	DF	P
t0	1	0.212	3	.	0.990	3	0.810
	2	0.286	3	.	0.930	3	0.490
t1	1	0.228	3	.	0.982	3	0.742
	2	0.262	3	.	0.956	3	0.596
t2	1	0.215	3	.	0.989	3	0.797
	2	0.269	3	.	0.949	3	0.565
t3	1	0.213	3	.	0.990	3	0.807
	2	0.287	3	.	0.930	3	0.487
t4	1	0.241	3	.	0.973	3	0.687
	2	0.272	3	.	0.947	3	0.556
t5	1	0.246	3	.	0.970	3	0.668
	2	0.272	3	.	0.946	3	0.553
t6	1	0.216	3	.	0.988	3	0.794
	2	0.257	3	.	0.961	3	0.620
t7	1	0.226	3	.	0.983	3	0.752
	2	0.276	3	.	0.942	3	0.535
t8	1	0.201	3	.	0.995	3	0.858
	2	0.270	3	.	0.949	3	0.565
t9	1	0.219	3	.	0.987	3	0.782
	2	0.253	3	.	0.965	3	0.639

[a] Rilley's significance correction

Abbreviations: DF, degree of freedom

Supplementary Table S6. Mauchly's sphericity test

Intrinsic effect	DF	Approximate square	DF	P	Epsilon[b]		
					Greenhouse- Geissler	Xin-ferdt	lower-bound
Time	0.000		44	.	0.294	1	0.111

We test the null hypothesis that the error covariance matrix of the dependent variable after an orthogonal transformation would be proportional to the identity matrix.

a. Design: [: cutoff:

b. Degree of freedom that can be used to adjust the mean significance test. The correction test is displayed in the within-subject effect test table.

Abbreviations: DF, degree of freedom

Supplementary Table S7. Within-subject effect test

		Sum of the squares III	DF	Mean square	F	P	Eta^2
Time	spherical degree	11686.933	9	512.511	1298.548	5.627	0
	Greenhouse	11686.933	2.648	1286.191	4414.264	5.627	0.017
	Xin-ferdt	11686.933	9	516.838	1298.548	5.627	0
	lower-bound	11686.933	1	2562.556	11686.93	5.627	0.077
Time * group	spherical degree	3859.267	9	291.444	428.807	1.858	0.091
	Greenhouse	3859.267	2.648	731.405	1457.681	1.858	0.2
	Xin-ferdt	3859.267	9	293.905	428.807	1.858	0.091
	lower-bound	3859.267	1	1457.222	3859.267	1.858	0.245
Deviation (time)	spherical degree	8307.6	36	241.361	230.767		
	Greenhouse	8307.6	10.59	605.717	784.465		
	Xin-ferdt	8307.6	36	243.399	230.767		
	lower-bound	8307.6	4	1206.806	2076.9		

degree of freedom

Supplementary Table S8. Pair comparison

Time	(I) Group	(J) Group	(I-J)	SD	P	95% CI	
						C1	C2
1	1	2	-218.667	261.978	0.451	-946.033	508.7
	2	1	218.667	261.978	0.451	-508.7	946.033
2	1	2	-267	275.068	0.387	-1030.713	496.713
	2	1	267	275.068	0.387	-496.713	1030.713
3	1	2	-230.333	270.391	0.442	-981.06	520.393
	2	1	230.333	270.391	0.442	-520.393	981.06
4	1	2	-236	261.894	0.418	-963.133	491.133
	2	1	236	261.894	0.418	-491.133	963.133
5	1	2	-221.667	257.495	0.438	-936.588	493.255
	2	1	221.667	257.495	0.438	-493.255	936.588
6	1	2	-244	262.79	0.406	-973.622	485.622
	2	1	244	262.79	0.406	-485.622	973.622
7	1	2	-221.667	263.575	0.448	-953.468	510.135
	2	1	221.667	263.575	0.448	-510.135	953.468
8	1	2	-209.333	266.905	0.477	-950.381	531.715
	2	1	209.333	266.905	0.477	-531.715	950.381
9	1	2	-248.667	280.726	0.426	-1028.087	530.754
	2	1	248.667	280.726	0.426	-530.754	1028.087
10	1	2	-236.667	276.041	0.44	-1003.081	529.747
	2	1	236.667	276.041	0.44	-529.747	1003.081

a) Multiple comparison regulation: Bonferroni method.
Abbreviations: SD, standard deviation; CI, confidence interval
