# Supplementary figures and images for: Evaluation and Limitations of the Novel Chemiluminescent Enzyme Immunoassay Technique for Measuring Total Tau Protein in the Cerebrospinal Fluid of Patients with Human Prion Disease: A 10-Year Prospective Study (2011–2020)
Source: Diagnostics (Basel). 2024 Jul 15;14(14):1520. doi: 10.3390/diagnostics14141520 (PMC11275853; doi:10.3390/diagnostics14141520)

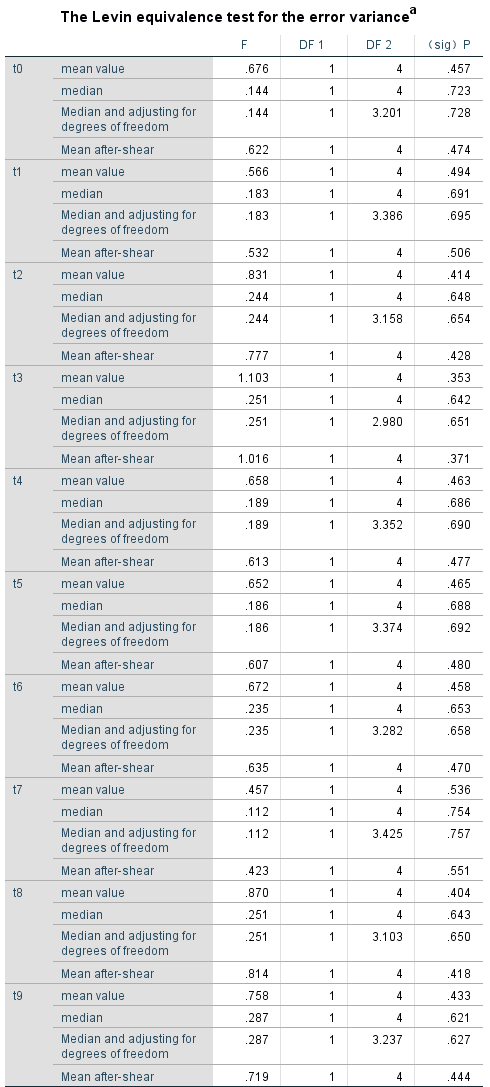

Supplement: Supplementary file 1 [file diagnostics-14-01520-s001.zip › Raw data S4.png]

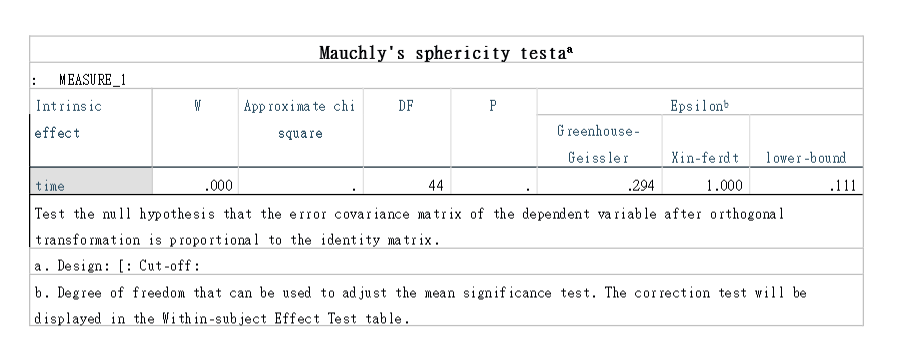

Supplement: Supplementary file 1 [file diagnostics-14-01520-s001.zip › Raw data S5.png]

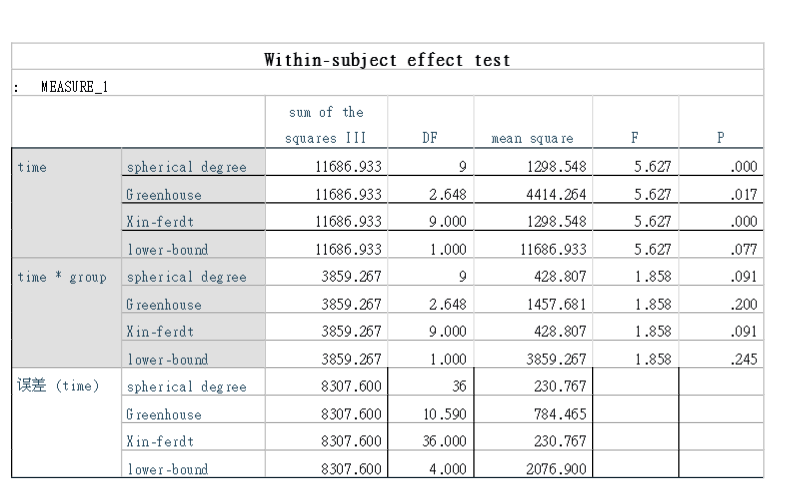

Supplement: Supplementary file 1 [file diagnostics-14-01520-s001.zip › Raw data S6.png]

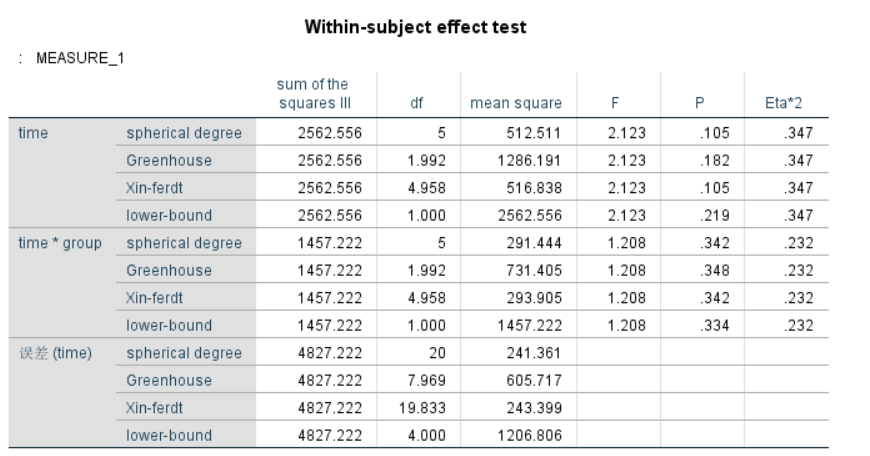

Supplement: Supplementary file 1 [file diagnostics-14-01520-s001.zip › Raw data S7.png]

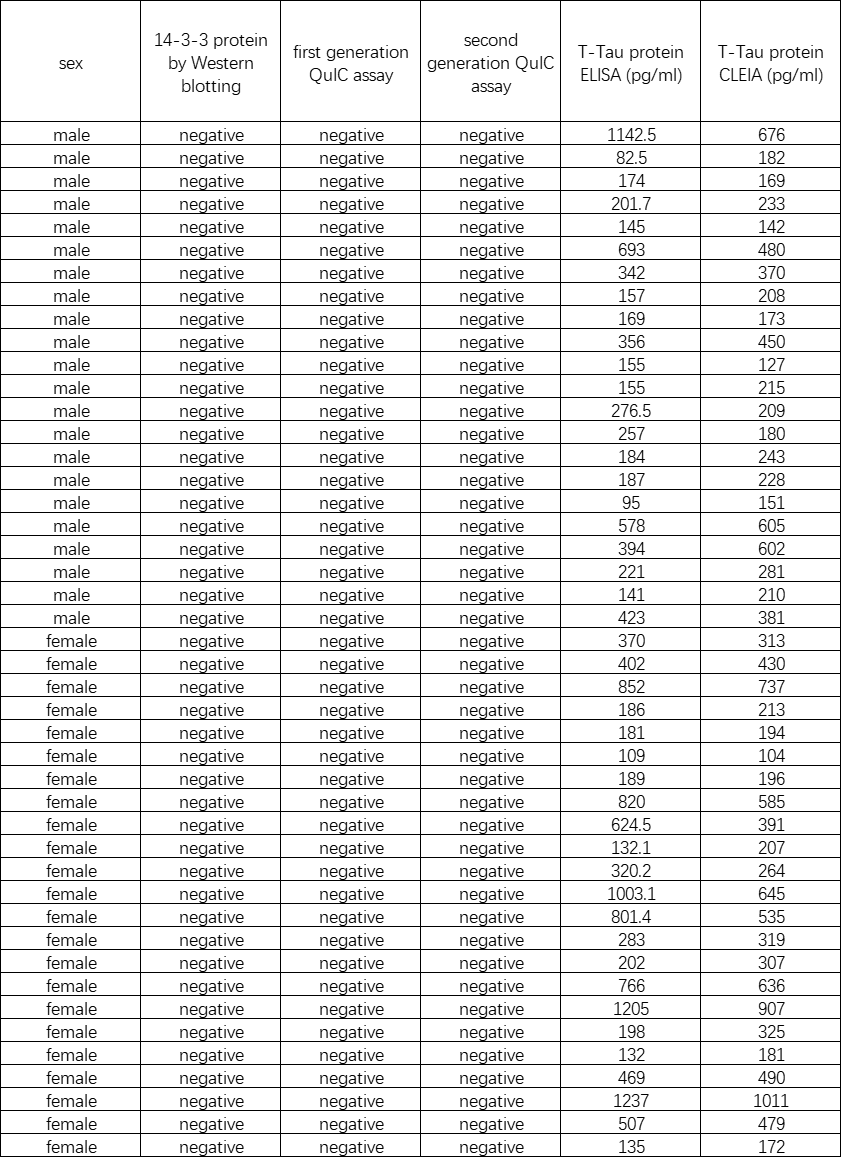

Supplement: Supplementary file 1 [file diagnostics-14-01520-s001.zip › Raw data S8.png]

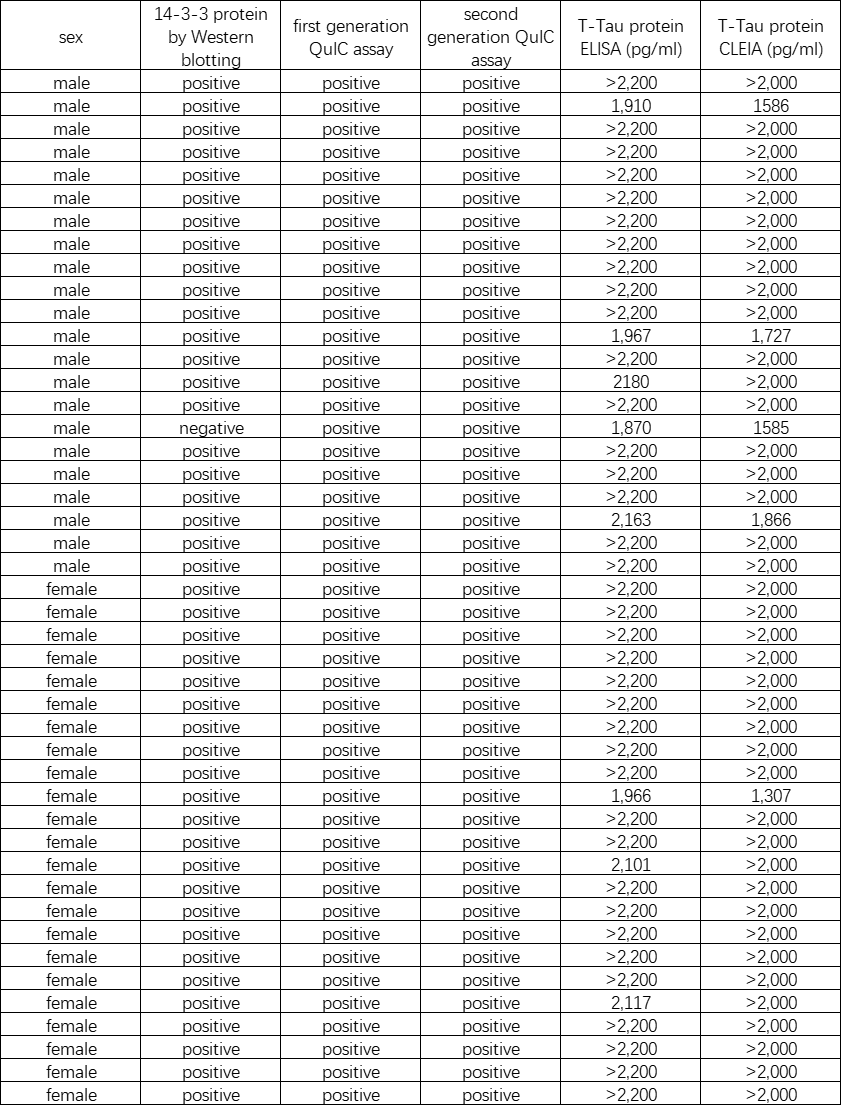

Supplement: Supplementary file 1 [file diagnostics-14-01520-s001.zip › Raw data S9.png]

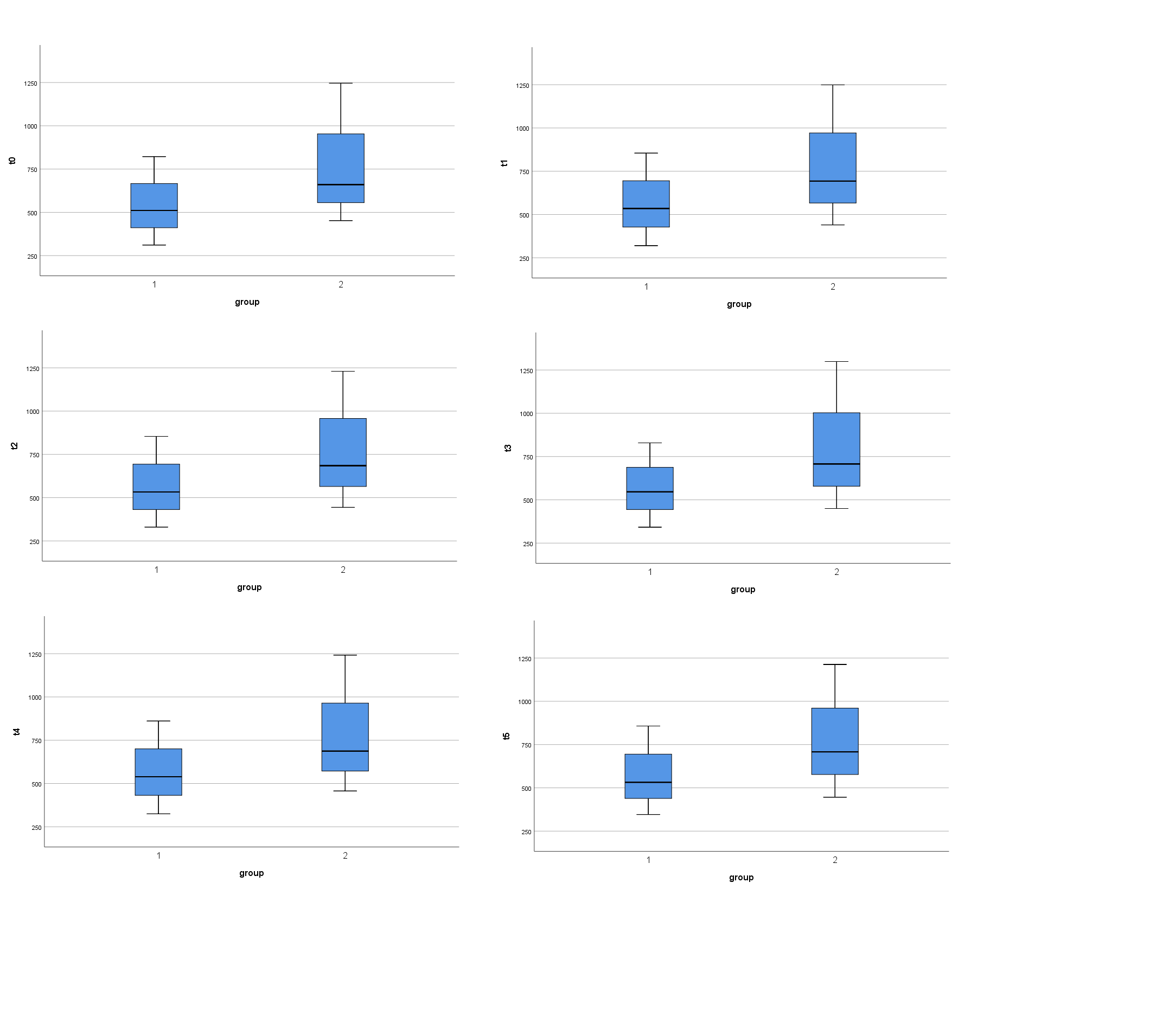

Supplement: Supplementary file 1 [file diagnostics-14-01520-s001.zip › Supplementary Figure S1.png]

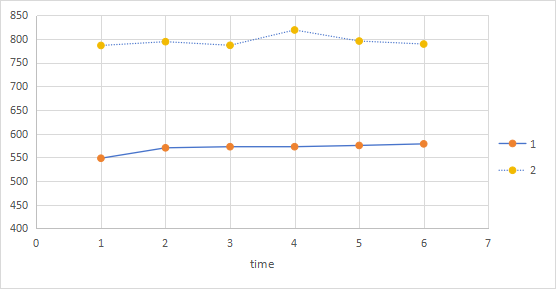

Supplement: Supplementary file 1 [file diagnostics-14-01520-s001.zip › Supplementary Figure S2.png]

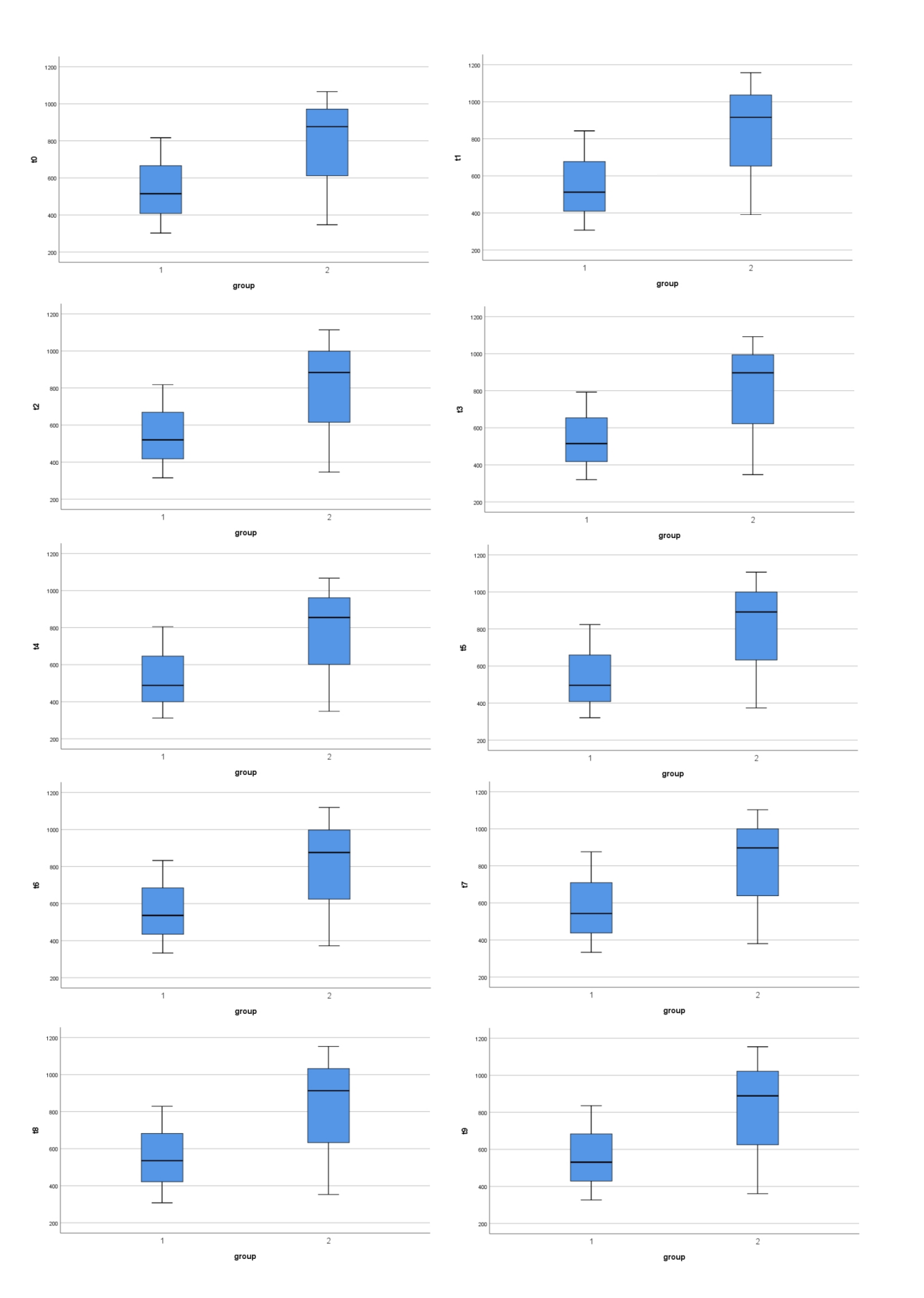

Supplement: Supplementary file 1 [file diagnostics-14-01520-s001.zip › Supplementary Figure S3.png]

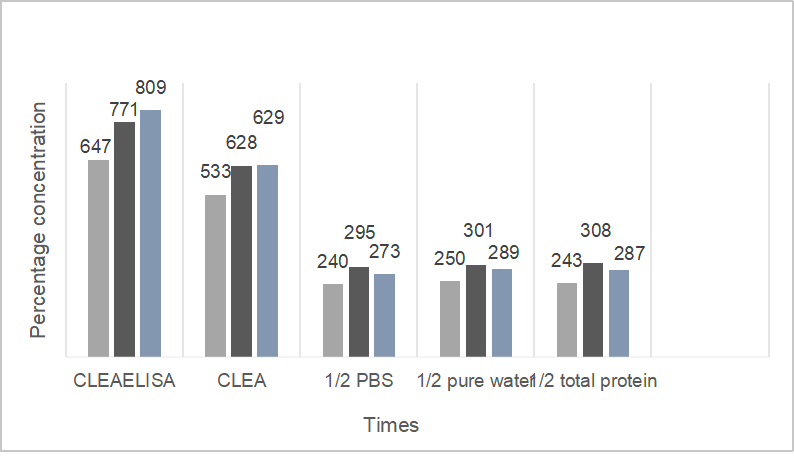

Supplement: Supplementary file 1 [file diagnostics-14-01520-s001.zip › Supplementary Figure S4a.png]

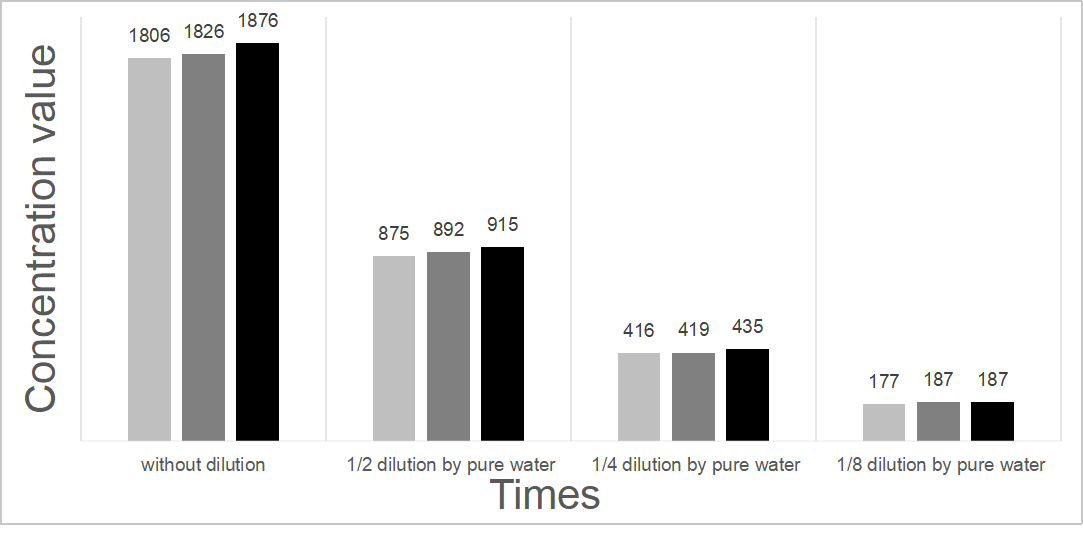

Supplement: Supplementary file 1 [file diagnostics-14-01520-s001.zip › Supplementary Figure S4b.png]
